# Distribution, Microfabric, and Geochemical Characteristics of Siliceous Rocks in Central Orogenic Belt, China: Implications for a Hydrothermal Sedimentation Model

**DOI:** 10.1155/2014/780910

**Published:** 2014-07-22

**Authors:** Hongzhong Li, Mingguo Zhai, Lianchang Zhang, Le Gao, Zhijun Yang, Yongzhang Zhou, Junguo He, Jin Liang, Liuyu Zhou, Panagiotis Ch. Voudouris

**Affiliations:** ^1^Key Laboratory of Mineral Resources, Institute of Geology and Geophysics, Chinese Academy of Sciences, Beijing 100029, China; ^2^Guangdong Provincial Key Lab of Geological Processes and Mineral Resource Survey, Guangzhou 510275, China; ^3^Department of the Earth Sciences, Sun YAT-SEN University, Guangzhou 510275, China; ^4^Department of Mineralogy-Petrology, University of Athens, Athens 15784, Greece

## Abstract

Marine siliceous rocks are widely distributed in the central orogenic belt (COB) of China and have a close connection to the geological evolution and metallogenesis. They display periodic distributions from Mesoproterozoic to Jurassic with positive peaks in the Mesoproterozoic, Cambrian—Ordovician, and Carboniferous—Permian and their deposition is enhanced by the tensional geological settings. The compressional regimes during the Jinning, Caledonian, Hercynian, Indosinian, and Yanshanian orogenies resulted in sudden descent in their distribution. The siliceous rocks of the Bafangshan-Erlihe ore deposit include authigenic quartz, syn-depositional metal sulphides, and scattered carbonate minerals. Their SiO_2_ content (71.08–95.30%), Ba (42.45–503.0 ppm), and ΣREE (3.28–19.75 ppm) suggest a hydrothermal sedimentation origin. As evidenced by the Al/(Al + Fe + Mn), Sc/Th, (La/Yb)_*N*_, and (La/Ce)_*N*_ ratios and *δ*Ce values, the studied siliceous rocks were deposited in a marginal sea basin of a limited ocean. We suggest that the Bafangshan-Erlihe area experienced high- and low-temperature stages of hydrothermal activities. The hydrothermal sediments of the former stage include metal sulphides and silica, while the latter was mainly composed of silica. Despite the hydrothermal sedimentation of the siliceous rocks, minor terrigenous input, magmatism, and biological activity partly contributed to geochemical features deviating from the typical hydrothermal characteristics.

## 1. Introduction

Siliceous rocks are widely distributed in the central orogenic belt (COB), China, and display a close relationship with the hydrothermal metallogenesis. In the COB, the distribution of siliceous rocks records the tectonic evolution of the whole orogenic belt [1–4]. It has been previously suggested that the siliceous rocks were originated from hydrothermal precipitation having a close relationship with the metallogenesis of many large hydrothermal ore deposits in China [5–7], especially those belonging to the SEDEX-type [8, 9]. The siliceous strata in the above hydrothermal ore deposits in China are either ore-hosting rocks or country rocks [1, 10–15].

The siliceous rocks of the COB and their relationship with the tectonic evolution and metallogenesis are still poorly described. According to [16], the distribution characteristics of siliceous rocks in the tectonic belts between two blocks have a close relationship with the tectonic evolution, inner dynamic, and metallogenesis [16]. The relationship between siliceous rocks and their associated orebodies has been discussed by [1, 4, 12, 17]. In addition, there are hydrothermal ore deposits in the COB, whose associated siliceous rocks are of hydrothermal genesis [1, 4, 17], but these ore deposits are regarded to be of orogenic type and formed during the orogeny [12]. As one of the typical hydrothermal ore deposits in the COB, the Bafangshan-Erlihe SEDEX deposit is a strata-bound Cu-Pb-Zn deposit with siliceous strata hosting and surrounding ores [5]. The siliceous rocks and associated mineralisation are slightly modified during subsequent orogenic processes [15, 18, 19], resulting in redistribution of metal sulphides in fissures, as commonly observed in orogenic type deposits [20]. The characteristics of Bafangshan-Erlihe ore deposit fit to both hydrothermal sedimentary ore deposits [11] and orogenic ore deposits [12], but no obvious evidence can directly exclude metallogenic sources from either hydrothermal seafloor (and subseafloor) precipitation or deposition from orogenic fluids. Thus, the distribution and compositional characteristics of the siliceous rocks may help clarifying the geological evolution and metallogenesis in the area.

The Bafangshan-Erlihe SEDEX deposit is of importance to understand the mechanism of hydrothermal water evolution in the paleo-ocean. It is located in the Fengxian-Taibai (or Fengtai) area [5], and is characterized by close relationships between the metallogenesis and deposition of siliceous strata. Previous work [1, 4] considered the siliceous rocks of the Bafangshan-Erlihe ore deposit to be of hydrothermal genesis, however ignoring the mineralogical characteristics, evolution mechanism of the hydrothermal water, and relationship between these siliceous rocks and their associated metal sulphides. In addition, the mechanism of hydrothermal convection necessary for the deposition of the siliceous rocks remained uncertain, and whether there are similarities to the published literature [21, 22] has to be confirmed. The aim of this paper is to study the characteristics of temporal and spatial distribution of the siliceous rocks in the COB, their microfabric and geochemical characteristics, and to present a model that incorporates the contribution of different geological processes during the deposition of hydrothermal silica and sulphide.

## 2. Geological Setting and Petrological Characteristics

### 2.1. Geological Setting

**Figure 1:**
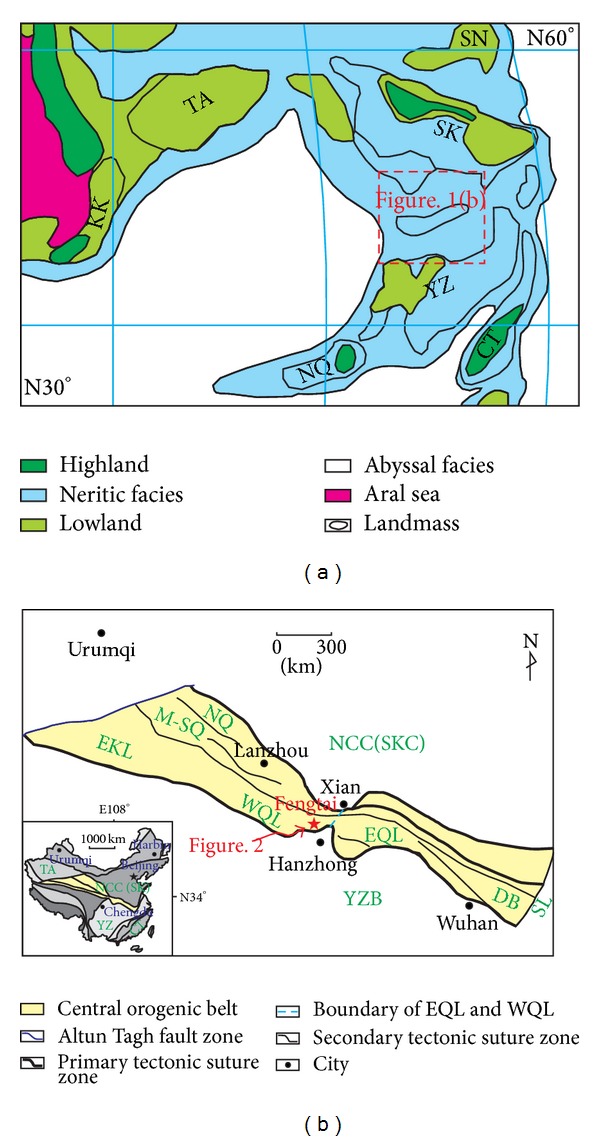
Geographical map and geological sketch of COB and the associated area ((a)—reconstruction map of the Middle-Permian paleo-continents [28]; (b)—geological framework of central orogenic belt [29, 30], TA: Tarim, KK: Karakum, SN: Sonnen, SK: Sino-Korean, YZ: Yangtze, NQ: Northern Qiangtang, CT: Cathaysian, EKL: Eastern Kunlun, M-SQ: Middle-South Qilian, NQ: North Qilian, WQL: Western Qinling, EQL: Eastern Qinling, SKC: Sino-Korean craton, YZB: Yangtze block, and DB:Dabie orogenic belt).

The COB has a complex tectonic evolution evidenced by the diversity in the eastern and western Qinling orogenic belts. The mainland of the present China is originated from the matching of several Neopaleozoic landmasses (Figure 1(a)). These blocks are Sino-Korean, Sonnen, Northern Qiangtang, Cathaysian, Tarim, Yangtze, and so forth. The COB is located in the middle of China and separated the Sino-Korean craton and Yangtze block on its north and south (Figure 1(b)). This orogenic belt is mainly made up of Kunlun, Qilian, Qinling, Dabie, and Sulu orogenic belts and passes through the provinces of Qinghai, Gansu, Shanxi, Henan, and Anhui, China, in northwest direction (Figure 1(b)). The main feature of the COB is the Qinling collisional orogenic belt [23]. The geological evolution of the Qingling orogenic belt can be divided into the formation of Precambrian basement from Neoarchaean to Mesoproterozoic (Ar_3_-Pt_2_), evolution of plate tectonics from Neoproterozoic to Middle Triassic (Pt_3_-T_2_), and intracontinental orogeny from Mesozoic (Late Triassic) to Cenozoic (Mz(T_3_)-Kz) [24]. Although the whole ocean basin of western Qinling starts from the Early Cambrian rift basin and ends up with the Triassic orogeny [25], the tectonic diversity of geological evolution contributed to the subdivision of a western section and an eastern section within the Qinling orogenic belt [26, 27]. The three tectonic cycles of opening-closing for the western Qinling are Early Cambrian ~ Early Devonian, Middle Devonian ~ Late Carboniferous, and Early Permian ~ Late Triassic [27], while the two tectonic phases of opening-closing for eastern Qinling are Early Cambrian ~ Late Silurian and Early Devonian ~ Late Triassic [26]. It is considered that the ocean basin of the COB is neritic in the Middle Permian [28], but whether there is a similar geographical pattern in the Devonian will be examined through the present study of the siliceous rocks from the Fengtai area.

**Figure 2:**
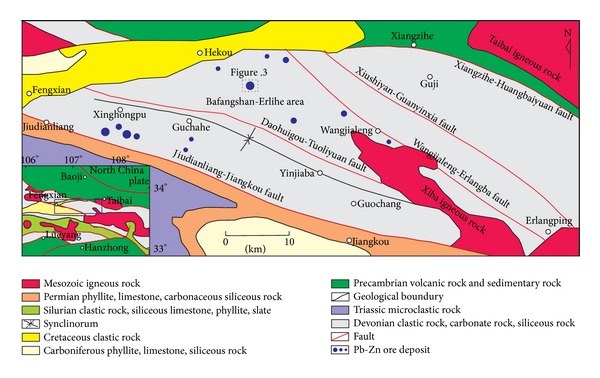
Geological sketch-map of the Fengxian-Taibai ore concentration area (after-[33]).

The Fengtai metallogenic area is located in the central part of the western Qinling orogenic belt. In the Fengtai metallogenic area (Figure 2), there are many polymetallic ore deposits, in the Bafangshan-Erlihe, Bijiashan, Dengjiashan, Qiandongshan, Yinmusi, Shoubanya, and Fengya areas. The tectonic frame is trending in NW direction as expressed by the Guchahe-Yinjiaba multiple downfolds and large faults (e.g., Xiushiyan-Guanyinxia, Wangjialeng-Erlangba, and Daohuigou-Tuoliyuan faults). The Fengtai area is located in the pull-apart basin of the northern region of Qinling microplate, whose southern and northern boundaries are Liuba peel thrust faults and the Shangzhou-Danfeng fault, respectively. Additionally, it is a secondary basin controlled by cross-basin synfaults [31], which are named Fengxian-Fengzhen-Shanyang and Jiudianliang-Zhenan-Banyanzhen faults [1, 32].

There is a diversity of sedimentary strata in the Fengtai area. The siliceous rocks associated with the metallogenesis of the Bafangshan-Erlihe ore deposit are mainly concentrated in the Middle-Upper Devonian strata (Figure 2). The Middle-Upper Devonian strata include clastic rocks of the Wangjialeng Formation, carbonate rocks of the Gudaoling Formation, metamorphic debris with interlayers of carbonate rocks of the Xinghongpu Formation, and fine-grained clastic rocks of Jiuliping Formation.

### 2.2. Geology of Ore Deposit

**Figure 3:**
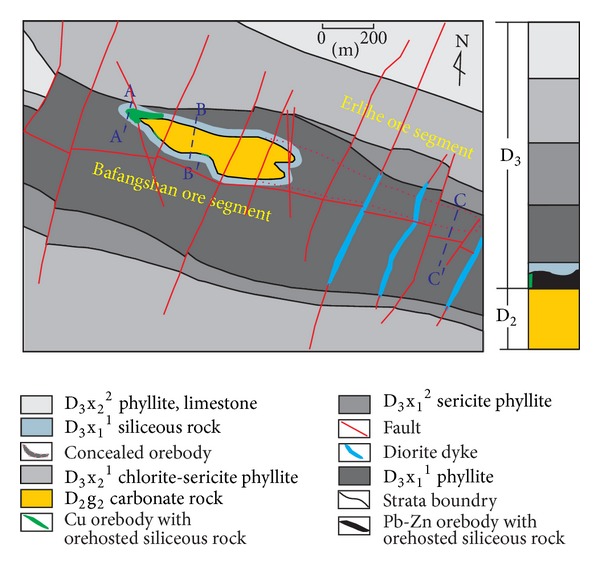
Geological map of the Bafangshan-Erlihe area (after-[33]).

The Bafangshan-Erlihe ore deposit is one of the significant hydrothermal ore deposits in the Fengtai metallogenic area located in the northwestern Fengtai basin (Figure 1). The strata belong to the Middle Devonian Gudaoling Formation (D2) and to the Upper Devonian Xinghongpu Formation D3 (Figure 3).

The Upper Devonian Xinghongpu Formation is made up of clastic rocks (siltstones and sandstones) with some foliated limestones [32, 34]. The strata of the Xinghongpu Formation are divided into three different lithological sections as follows: the first section of the Xinghongpu Formation includes arenaceous phyllite; the second section of the Xinghongpu Formation is comprised of carbon-bearing phyllite and sericitic phyllite; the third section is mainly composed of banded lamellar limestone and calcitic-sericitic-phyllite. In the bottom of the first section, there are ferrodolomite, sericitic phyllite, and siliceous rocks, which were the country rocks of the Cu-Pb-Zn orebody.

The Middle Devonian Gudaoling Formation occupies the entire Bafangshan-Erlihe ore deposit (Figure 3). This formation extends downward to the lower part, beneath the earth's surface, to make up the core of the Bafangshan-Erlihe anticlinal. This formation is divided into two parts: the first part is a clastic series including sandstones and shales and the second part is composed of carbonate rocks.

The hydrothermal sedimentary sequence is located in the interface between the Middle Devonian Gudaoling Formation and the Upper Devonian Xinghongpu Formation. This sedimentary sequence is composed of siliceous rocks, siliceous ferrodolomites, silicified limestones, and limestones. The stratum of the siliceous rocks, ranging from 1 m to 30 m, belongs to the ore-bearing strata. There are boudinaged siliceous rocks and aggregates of siliceous ankerite in the ore-bearing strata. The siliceous rocks are exposed on the surface at the Bafangshan area whereas they extend downwards to the Erlihe area. In addition, some of the orebodies are hosted in limestones or ferrodolomites.

The Bafangshan-Erlihe ore region is intensively folded and faulted (Figure 3). The Cu-Pb-Zn orebodies are controlled by the Bafangshan-Erlihe anticline. There are two types of faults: the normal faults, striking NNE-SSW and NNW-SSE, are widely distributed across the study area with an angle of dip of 70°; the compression-shear faults, striking EW, with hades ranging from 48° to 73°. The area is characterized by a weak magmatic activity related to the emplacement of some dikes along NE-striking normal faults. The dykes include quartz-porphyries and quartz-diorite-porphyries.

### 2.3. Distribution of Orebody

**Figure 4:**
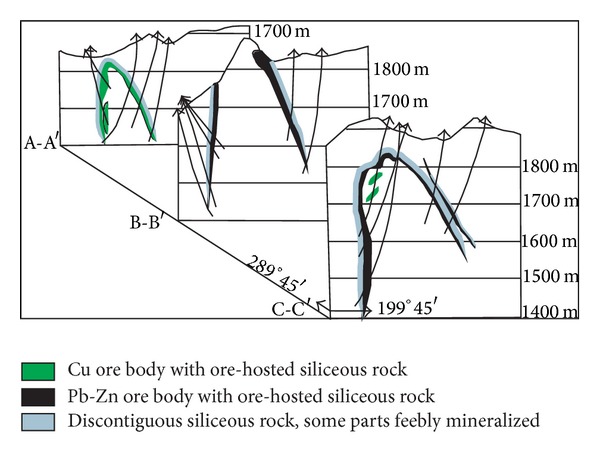
Cross-sections of ore body and siliceous rock in the Bafangshan-Erlihe Cu-Pb-Zn ore deposit. For location see Figure 3 (After-[12]).

The Cu-Pb-Zn ore deposits in the Bafangshan-Erlihe area are controlled by anticlines (Figure 3) [32, 34]. The Bafangshan-Erlihe ore deposit is divided into two segments, which are located in the Bafangshan and Erlihe areas. Because of denudation at the top of the anticlines, the orebodies of the Bafangshan segment are exposed at the surface. In the core of the anticlines, the ore strata are distributed with the shape of an irregular circle around the limestone. They stretch eastward and become underground toward the Erlihe ore area. In the Erlihe area, the orebodies are concealed at a depth of up to 800 meters. The proven orebodies are mostly developed on the arch bend of the anticlines in longitudinal profile (Figures 3 and 4). Orebodies are stratabound and generally contact with the adjoining country rocks conformably. The primary orebody is consistent with the construction of the anticlines, and it is more than 2300 meters in length and ranges from 100 meters to 300 meters in width [34]. On the incline, the primary orebodies stretch downwards and become narrower from east to west.

There are obvious horizontal and vertical mineralization characteristics (Figure 4). At the Bafangshan ore deposit the ore grade decreases with depth [34, 35], thus, all copper, lead and zinc are distributed in the upper part of the deposit. Along strike, the Bafangshan ore deposit generally extends from east to west and can be grouped into the east, middle, and west parts without clear mineralization boundaries between them [35]. The eastern part is characterized by mineralised zones of copper, lead and zinc, dominated by chalcopyrite, sphalerite, and galena, respectively. The middle part is mainly composed of lead and zinc sulphides (e.g. galena and sphalerite). The main mineralization in the western part consists of chalcopyrite with traces of galena and sphalerite.

### 2.4. Petrological Characteristics

**Figure 5:**
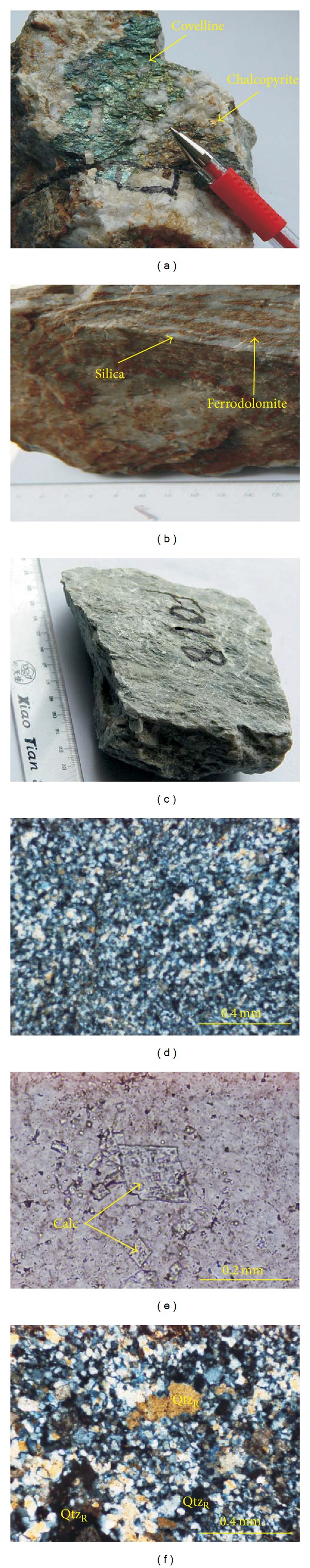
Ores and siliceous rocks from the Bafangshan-Erlihe ore deposit ((a) copper ore; (b) ferrodolomite-bearing siliceous rocks with lamellar structures, the lamellae of the ferrodolomite were oxidised; (c) pure siliceous rock; (d) finely crystalline siliceous rock under parallel nicols; (e) idiomorphic calcite in parallel nicols; (f) recrystallized quartz in crossed nicols; Calc-Calcite, Qtz_R_-recrystallized quartz).

In the Bafangshan-Erlihe ore deposit, mineralization is hosted in siliceous rock and ferrodolomite breccias. The breccias are cemented by chalcopyrite (Figure 5(a)), galena, sphalerite, and pyrite. Some of the ferrodolomite-bearing siliceous rocks are oxidised and display alternating layers of siliceous rocks and ferrodolomites (Figure 5(b)). The ores exhibit brecciated, stockwork, and lamellar structures. The pure siliceous rocks, without mineralisation, are grey, compacted, and hard with brecciated, massive (Figure 5(c)), and banded structures (Figure 5(b)). Microscopically, the siliceous rocks are composed of finely crystalline quartz, forming close-packed or interlocking textures (Figure 5(d)), which are similar to slightly recrystallized siliceous rocks of hydrothermal genesis [36]. Minor idiomorphic carbonate minerals are also present in the siliceous rocks (Figure 5(e)). In addition, there are also some recrystallized quartz grains with much larger grain sizes (Figure 5(f)), surrounded by the finely crystalline quartz grains in the matrix.

## 3. Samples and Experiments

During the pretreatment processes, fresh samples (including ore-hosting siliceous rocks and pure siliceous rocks without mineralization) of Bafangshan-Erlihe polymetallic ore deposit were selected, cleaned in ultrapure water, dried, and then divided into two groups, namely, one polished into thin sections (≤0.03 mm) and the other crushed into grains of 0.3 cm-equivalent spherical diameter in a clean corundum jaw-breaker. A subsample from the latter group was selected, cleaned, redried, and ground to 0.075 mm diameter particles in an agate ball mill (NO. XQN-500x4). Additionally, all the ore-hosting siliceous rocks were repeatedly separated from the ore to ensure their purification.

The pretreatment and analysis of each sample's major elements were carried out in Guilin's Research Institute of Geology and Mineral Resource Test Centre, and the results are shown in Table 1. The SiO_2_ content was analysed by potassium hexafluorosilicate titration to an accuracy of between 1% and 1.5%. The Al_2_O_3_ constituents were analysed with ultraviolet spectrophotometry (to contents below 1% by mass, using instrument type UV-120-02, at an accuracy of 0.5% to 1%) or by EDTA titration (for contents above 1%, to an accuracy of 1.5%). The Na_2_O, K_2_O, and MgO constituents were analysed by atomic absorption spectroscopy (instrument type HITACHI Z-5000, to an accuracy of 1%). The CaO was analysed by atomic absorption spectroscopy (for contents below 10%, using a HITACHIZ-5000 instrument, with an accuracy of 1%) or by EDTA titration method (for contents above 10%, at an accuracy of 1.5%). The P_2_O_5_ was analysed by ultraviolet spectrophotometry (for contents below 1%, using instrument type UV-120-02, with an accuracy of between 0.5% and 1%). The TiO_2_ and MnO constituents were analysed by inductively coupled plasma optical emission spectrometry (ICP-OES) with instruments iCAP_6300_RADIAL and iCAP_6301_RADIAL, with accuracies between 0.5% and 1.5%. The FeO and Fe_2_O_3_ contents were obtained by potassium dichromate titration.

An inductively coupled plasma mass spectrometer (ICP-MS, Instrument Model: PE Elan6000) with an analytical accuracy of 1% to 3% was used to test for trace and rare earth elements. The test solution was prepared by acid-soluble dissolution and the experiment was performed in accordance with standard protocols. Samples weighing 100 mg were placed in a sealed Teflon container and l mL of concentrated HF and 0.3 mL of 1 : 1 HNO_3_ were added. Following ultrasonic oscillation, the samples were placed on a hot plate at 150°C and then evaporated to dryness, remixed with the same amount of HF and HNO_3_, and heated under confinement for a week (at approximately 100°C). After evaporation and dissolution in 2 mL of 1 : 1 nitric acid, the sample was added to an Rh internal standard, diluted to 1/2000th of its original concentration, and tested in the PE Elan6000 ICP-MS.

Pretreatment for Raman spectroscopy and X-ray diffraction (XRD) analyses was performed in the Guangdong Provincial Key Laboratory of Geological Processes and Mineral Resource Survey. Raman analysis was performed in the State Key Laboratory of Geological Processes and Mineral Resources, where the Renishaw RM-1000 invia microconfocal instrument was used for the Raman experiments, with excitation by the 514.5 nm line of an Ar^+^ laser. Raman spectra were recorded to a resolution of 1 cm^−1^ from 50 cm^−1^ to 1800 cm^−1^. An XRD analysis was carried out in the laboratory of the College of Chemistry and Chemical Engineering of Sun Yat-Sen University. XRD data were collected with an X-ray powder diffractometer (instrument type: D/Max-2200 vpc) in reflection focusing geometry mode (Cu K*α* radiation; 40 kV/30 mA, scanning speed: 0.12 s* per *step, step length: 0.02°, continuous scanning mode). Over a range of 5° ≤ scanning angle ≤ 100°, the data were postprocessed by JADE-5.0 software based on the eight highest peaks for the identification of several mineral types.

The Scanning Electron Microscope (SEM) and Energy Disperse Spectroscopy (EDS) analysis were carried out by the State Key Laboratory of China's University of Geosciences. The ring scanning electron microscopy instrument was a Quanta 200F environmental scanning electron microscopy-energy spectrum-electron backscatter diffraction system (SEM-EDS) with a resolution of 3.5 nm and a magnification of 7 to 1,000,000.

## 4. Analytical Results

### 4.1. Distribution Characteristics

The COB, trending in a NW direction, mainly includes the provinces of Qinghai, Gansu, Shanxi, Henan, and Anhui, China (Figure 1(b)). In the present study, the numbers and localities of siliceous rocks were calculated from these five provinces based on location and formation as the main parameters. The localities of the siliceous rocks were calculated from the regional geology of Zhejiang, Jiangxi, Hunan, Guangdong, and Guangxi provinces [32, 37–40]. In locations, where there is a uniform distribution of siliceous rocks within diverse strata, these siliceous rocks were separated on the basis of Formation unit. Additionally, the Precambrian strata were divided into Mesoproterozoic and Neoproterozoic (Sinian) strata according to the literatures [32, 37–40].

#### 4.1.1. Temporal Distribution

**Figure 6:**
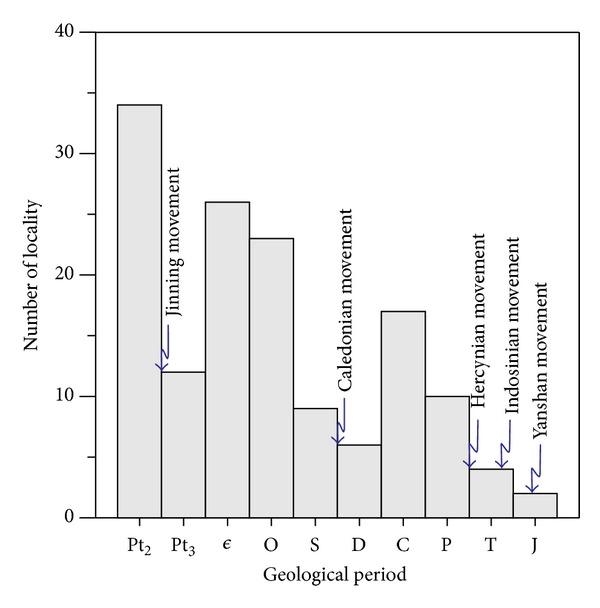
Column diagram of number for localities of siliceous rocks in the COB (data were calculated from literatures [32, 37–40]).

In the COB, the marine siliceous rocks display a periodic quantitative distribution from Mesoproterozoic to Jurassic. There were positive peaks of their distribution number in the Mesoproterozoic, Cambrian ~ Ordovician, and Carboniferous ~ Permian (Figure 6). The highest distribution of siliceous rocks occurs during the Mesoproterozoic, possibly related with the sustained break-up of Columbia supercontinent with a relatively long geological history [41, 42]. The distribution numbers of siliceous rocks increased suddenly at the beginning of Cambrian, which quite agreed with the beginning of collapse of the Qinling orogenic belt [27]. Another sudden increase in their distribution number started from the beginning of Carboniferous, which agreed well with the extensional tectonic setting of the whole Qinling orogenic belt [26, 27]. From Mesoproterozoic to Jurassic, there were several sudden decreases in the distribution number of the siliceous rocks, which started from the beginning of Sinian, Permian, and Triassic, respectively. In the previous study [16, 37–40], the cratonisation of Rodinia continent took place between Mesoproterozoic and Neoproterozoic, and was contributed by the Jinning orogeny. The Caledonian orogeny took place in Late Silurian, in accordance with the decline in distribution number of siliceous rocks in Silurian. Additionally, there was a sudden decrease in their distribution number in Triassic, which is in accordance with the Hercynian orogeny at the end of Devonian. During the geological evolution of COB, the extensional tectonics of different tectonic cycles started from the beginning of Cambrian and Middle Devonian [26, 27], which quite agreed with the sudden increase in distribution numbers of siliceous rocks. Collision of continental plates during the Jinning, Caledonian and Hercynian movements, resulted in compressional regimes and a sudden decrease in distribution numbers of siliceous rocks. The siliceous rocks faded away since the Hercynian movements at the end of Permian, as well as in the following Indosinian and Yanshanian movements. The marine siliceous rocks disappeared since the end of Jurassic due to the regression of the whole COB. Thus, the geological setting was tensional in the tectonic eras of Caledonian (from Sinian to Late Silurian) and Hercynian (from Devonian to Late Permian) periods, when the siliceous rocks had the largest distribution number with the widest distribution. On contrary, the Jinning, Caledonian, Hercynian, Indosinian, and Yanshanian movements contributed to compressional setting and resulted in negative peaks of distribution numbers for the siliceous rocks with smaller distribution scale. According to this, the widest distributions of siliceous rocks agreed with the tensional setting, whereas the decreasing numbers of siliceous rock were attributed by the compressional settings. Previous studies show there is periodic distribution of the siliceous rocks in the Qinling orogenic belt [25], which quite agree with the present study. So, the siliceous rocks were preferential to develop more widely in tensional settings in the COB and decreased quantitatively due to the compressional settings.

#### 4.1.2. Spatial Distribution

**Figure 7:**
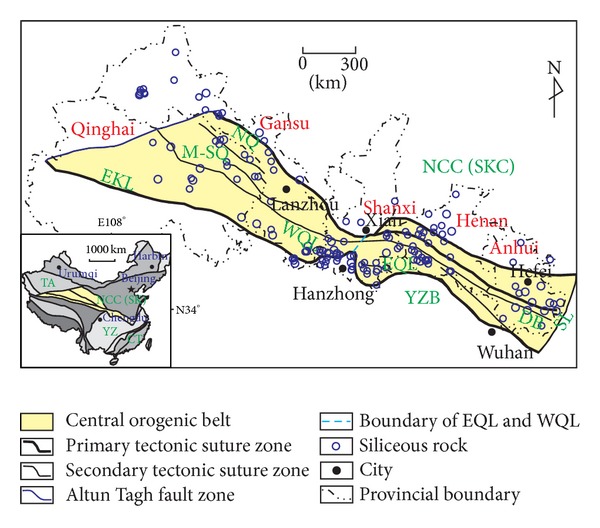
Spatial distribution of total siliceous rocks in the COB (EKL: eastern Kunlun; M-SQ: Middle-South Qilian; NQ: North Qilian; WQL: Western Qinling; EQL: Eastern Qinling; SKC: Sino-Korean craton; YZB: Yangtze block; DB: Dabie orogenic belt; Data sources: [32, 37–40]).

**Figure 8:**
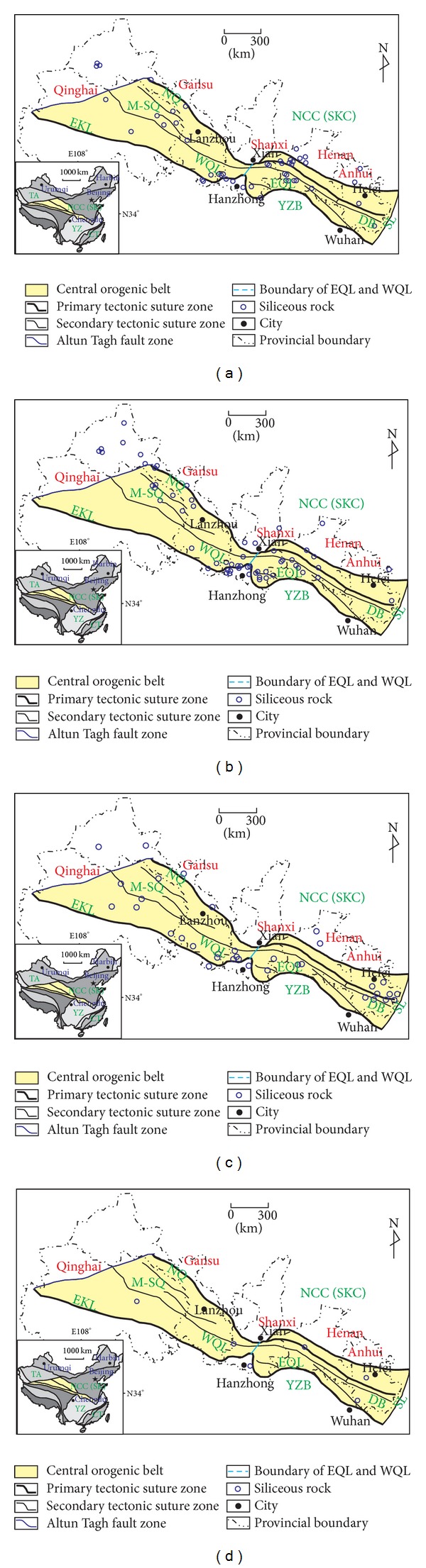
Distribution of siliceous rocks in central orogenic belt of China ((a): Precambrian; (b): Eopaleozoic; (c): Neopaleozoic; (d): Mesozoic; EKL: eastern Kunlun, M-SQ: Middle-South Qilian; NQ: North Qilian; WQL: Western Qinling; EQL: Eastern Qinling; SKC: Sino-Korean craton; YZB: Yangtze block; DB: Dabie orogenic belt; Data sources: [32, 37–40]).

The siliceous rocks were mainly located in the border area of suture zones (Figure 7). The siliceous rocks are widely distributed in the central area of China, mainly within the COB and its adjacent areas (Figure 7). Because there was a clear geological diversity between eastern and western Qinling orogenic belt [26, 27], the COB was divided into eastern section (including eastern Qinling and Dabie orogenic belts) and western section (including western Kunlun, Qilian, and Western Qinling orogenic belts). The siliceous rocks are widely distributed in the Precambrian strata of the COB without a clear diversity between eastern and western sections (Figure 8(a)). In the Eopaleozoic strata (Figure 8(b)), the siliceous rocks are extensively distributed mainly in the eastern COB. The distribution of Neopaleozoic siliceous rocks is relatively weak (Figure 8(c)) mainly in the western COB. Finally, the Mesozoic siliceous rocks was very weakly and sporadically distributed in both eastern and western sections (Figure 8(d)). According to the aforementioned, the siliceous rocks are variously distributed in time and space along the COB: they are concentrated along the suture zones, mainly extending in Caledonian and Hercynian strata in the eastern and western sections of COB respectively. During tectonic evolution, the suture zones underwent extensional stress with many normal faults facilitating magma emplacement and transport of hydrothermal fluids [16, 43]. In the COB, siliceous rocks with a more preponderant distribution became younger from the eastern section to the western section, which is possibly attributed to the compressional setting of the eastern section and tensional setting of the western section due to the Neopaleozoic expanding of the paleo-tethys ocean [27]. Additionally, the collisional orogeny during the Indosinian and Yanshanian movements contributed to compressional settings and therefore resulted in the quantitative reduce of siliceous rocks in the Mesozoic. In summary, the siliceous rocks were mainly developed in tensional settings with a preferential distribution next to the suture zones.

### 4.2. Major and Trace Elements

Geochemical information (e.g. major-, trace- and rare earth element data) of the analysed siliceous rocks from the Bafangshan-Erlihe ore deposit, as well as the data from [1], used to examine their sedimentary depositional environment, genesis and geological context, is summarized below.

**Figure 9:**
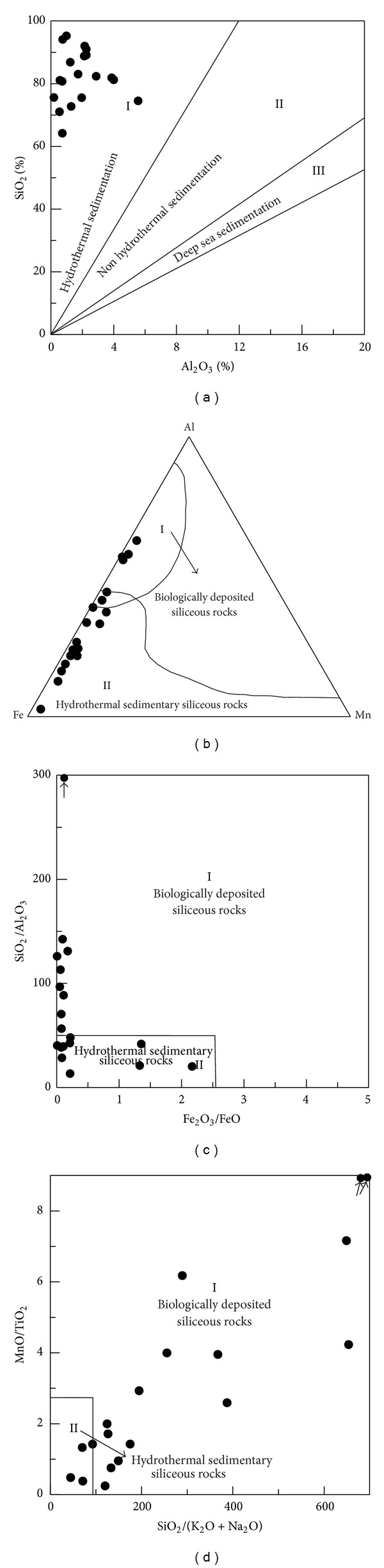
Major element discrimination diagrams supporting hydrothermal and biological processes for the genesis of siliceous rocks of the Bafangshan-Erlihe area (After: (a)—[51]; (b)—[52]; (c); (d)—[53]).

**Figure 10:**
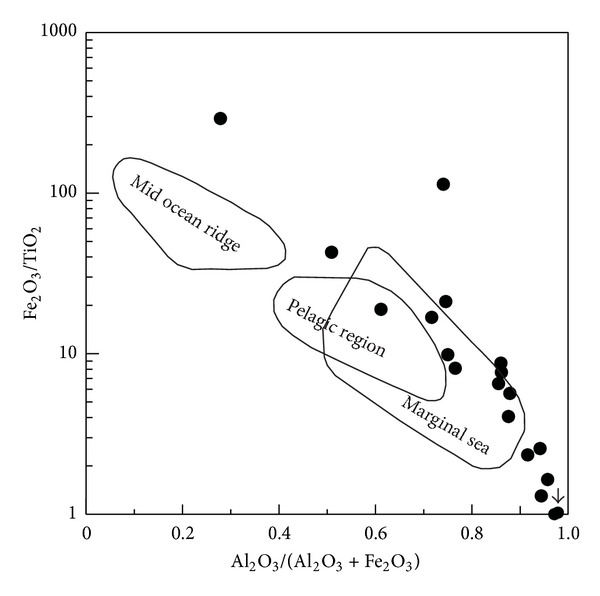
Fe_2_O_3_/TiO_2_ versus Al_2_O_3_/(Al_2_O_3_ + Fe_2_O_3_) discrimination diagram indicating the formation environment of the siliceous rocks of the Bafangshan-Erlihe area (After [53]).

**Figure 11:**
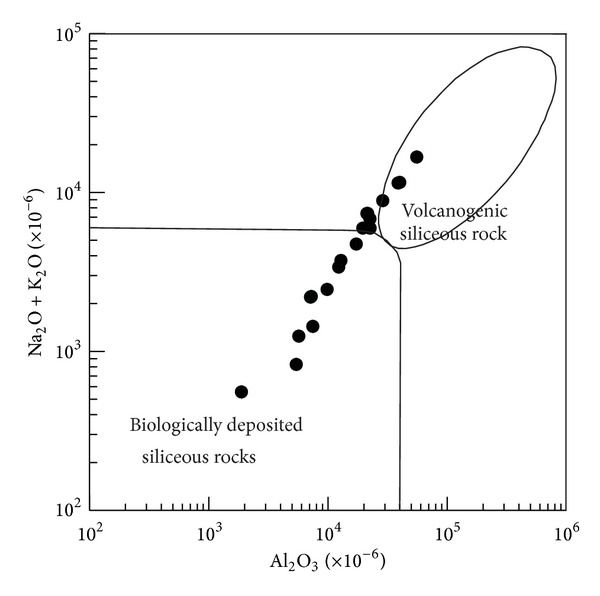
Major element discrimination diagram indicating biological and volcanic processes for the genesis of the Bafangshan-Erlihe area (After-[56]).

**Figure 12:**
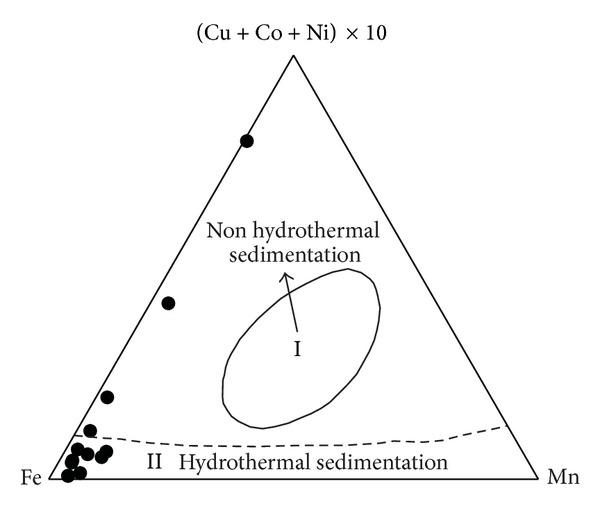
Trace element discrimination diagram indicating mostly hydrothermal genesis for siliceous rocks from the Bafangshan-Erlihe area (After-[63]).

**Figure 13:**
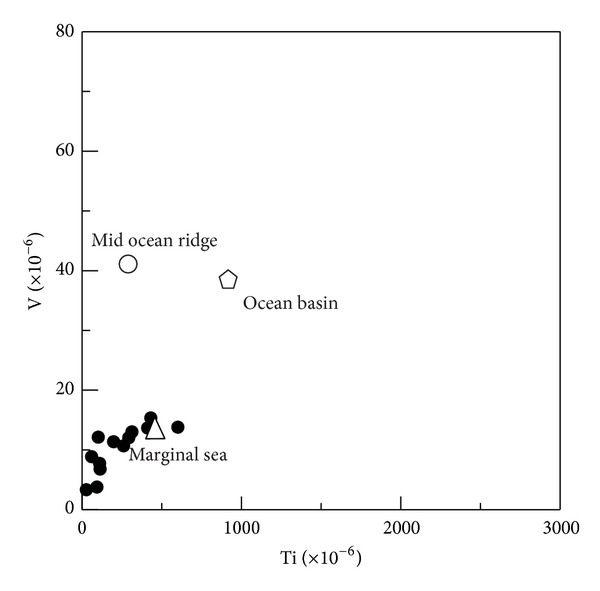
Trace element discrimination diagram demonstrating formation environment at a marginal sea basin for the siliceous rocks of the Bafangshan-Erlihe area (After-[46]).

**Figure 14:**
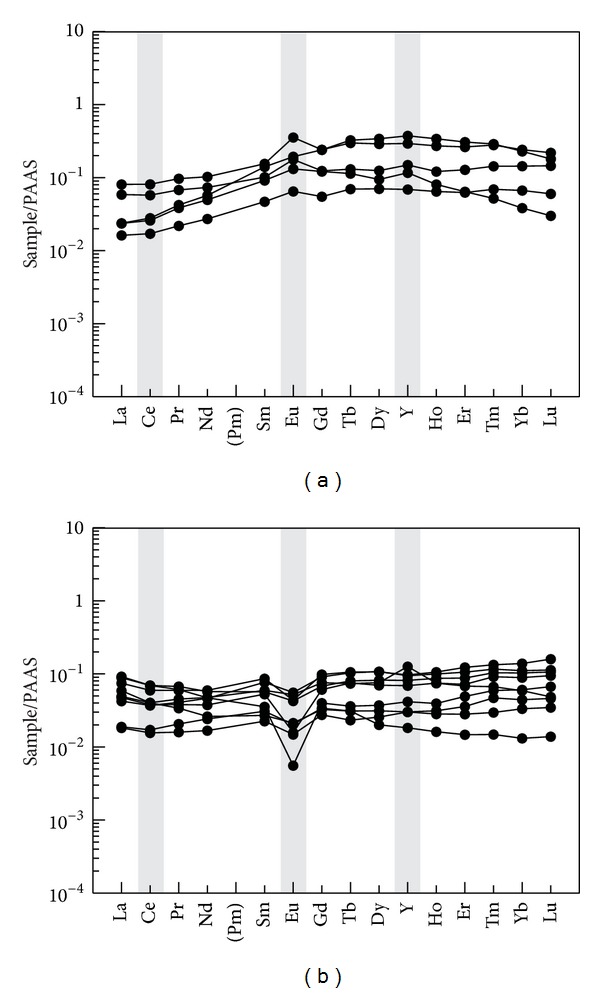
PAAS normalized REE pattern for siliceous rocks from the Bafangshan-Erlihe area.

**Figure 15:**
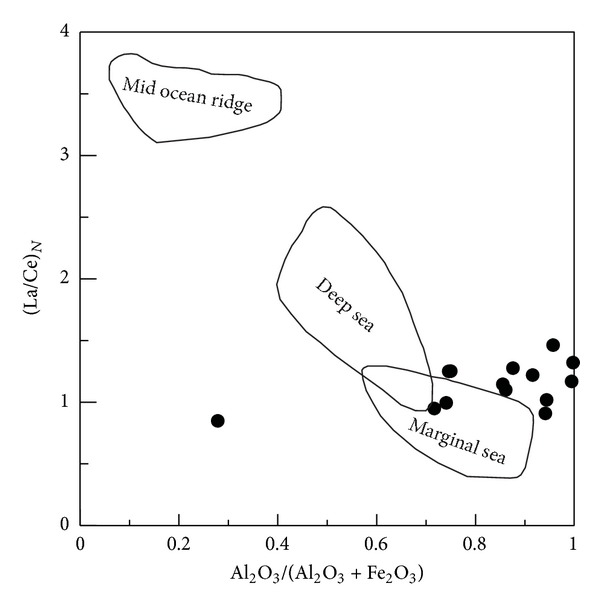
Al_2_O_3_/(Al_2_O_3_ + Fe_2_O_3_) − (La/Ce)_*N*_ discrimination diagram for siliceous rock of the Bafangshan-Erlihe area (After-[53]).

(1) The major elemental analysis results and geochemical indices for the siliceous rocks are listed in Tables 1 and 2. The geochemical characteristics of major elements indicated the following.

(a) A hydrothermal genesis of the siliceous rocks is suggested according to the major element characteristics with their formation process influenced by biological activities. The SiO_2_ content of the siliceous rocks ranges from 71.08% to 95.30% with an average of 84.10%. The Si/Al ratios range between 11.83 and 125.68, which are lower than that of pure siliceous rock with ratios usually in the range from 80 to 1400 [46]. The Al/(Al + Fe + Mn) ratios range from 0.03 to 0.58, which are consistent with that of typical hydrothermal siliceous rock of below 0.4 [47]. Taking into account that MgO is depleted in modern ocean ridges and was zero in the hydrothermal water at 350°C from the East Pacific [48], the MgO content of the analysed siliceous rocks (0.15 % to 2.88 %) is higher than that of pure hydrothermal siliceous rocks. In addition, the (Fe + Mn)/Ti ratios of the siliceous rock varies from 15.59 to 1031.45, which is consistent with that of typical hydrothermal sediments (higher than 20 ± 5 [49]). The Fe_2_O_3_/FeO ratios range from 0.01 to 2.17 and are lower than that of the siliceous rocks of hydrothermal genesis (typically 0.51 [50]). These characteristics, as well as the associated geochemical discrimination diagrams of Al_2_O_3_-SiO_2_ (Figure 9(a)) and Mn-Al-Fe (Figure 9(b)), strongly support their genesis by hydrothermal sedimentation. Moreover, the siliceous rocks plotted in the Fe_2_O_3_/FeO-SiO_2_/Al_2_O_3_ and SiO_2_/(K_2_O + Na_2_O)-MnO/TiO_2_ diagrams indicated biological influences during their formation processes (Figures 9(c) and 9(d)).

(b) The siliceous rocks of the Bafangshan-Erlihe ore deposit were formed in a marginal sea basin. According to [54], the Al/(A1 + Fe + Mn) ratios of siliceous rocks are diverse depending upon sedimentary processes, and they decrease from 0.619 in the continental margin to 0.319 in oceanic basins or islands, with a lower value 0.00819 at the mid-ocean ridge. This gradual reduction showed the progressive influences of hydrothermal precipitation. The Al/(Al + Fe + Mn) ratios of the studied siliceous rocks range from 0.03 to 0.59, which are approximately equal to that of siliceous rocks from ocean basins or continental margins. The contents of terrigenous Al and Ti are higher at the continental margin, and they decrease with distance from the marginal sea. The MnO/TiO_2_ ratios range from 0.25 to 45.98, which is higher than that of typical samples at the continental margin with ratios below 0.5 at the continental margin [52]. In addition, the Al/(Al + Fe) ratios range from 0.03 to 0.59, which is lower than that of the bedded siliceous rocks along the continental margin [48]. The Al_2_O_3_/(Al_2_O_3_ + Fe_2_O_3_) ratios range from 0.51 to 0.96, which is close to that of typical siliceous rock from marginal seas [53]. The above characteristics agree with the associated discrimination diagram (Figure 10).

(c) There was no obvious volcanic activity during the precipitation of hydrothermal silica. The K_2_O/Na_2_O ratios for the analysed siliceous rocks range from 0.64 to 23.63, which is much higher than that of the siliceous rocks deposited by submarine volcanism [48]. The SiO_2_/(K_2_O + Na_2_O) ratios of the siliceous rocks ranged from 44.51 to 856.39, which is much higher than that of the siliceous rocks from chemical deposition related to volcanic eruptions [55]. The SiO_2_/Al_2_O_3_ ratios and the SiO_2_/MgO ratios range from 13.42 to 142.58 and from 28.61 to 649.33 respectively, which is much higher than that of siliceous rock related to magmatism, with SiO_2_/Al_2_O_3_ < 13.7 [14]. The SiO_2_/MgO ratios range from 28.61 to 649.33, which is much higher than that of typical siliceous rocks related to magmatism [14]. Despite the fact, that, there was no obvious volcanic activity during their formation process, some analysed siliceous rocks plot in the category related to volcanism (in Figure 11). This may indicate a magmatic contribution related to the deep faults.

(2) The analytical results of trace elements are listed in Table 3. Trace element geochemistry indicates the following.

(a) The siliceous rocks were originated from hydrothermal precipitation. The Ba content of the siliceous rock ranges from 42.45 ppm to 503.00 ppm with an average of 196.64 ppm and is between that of the MORB (12.00 ppm [57, 58]) and the crustal rocks (707 ppm [59]). The U ranges from 0.07 ppm to 1.53 ppm, with an average of 0.48 ppm, which is also between that of the MORB (0.10 ppm [57, 58]) and the crustal rocks (1.30 ppm [59]). These values are similar to those of hydrothermal sedimentary siliceous rocks and contrast from those from terrestrial sediment [60]. The U/Th ratios range from 0.07 to 4.91, which is slightly lower than that of hydrothermal sediments [61]. The Ba/Sr ratios range from 0.14 to 25.97, which is in accordance with that of hydrothermal siliceous rocks (Ba/Sr > 1 [62]). Additionally, a hydrothermal genesis of the siliceous rocks is supported from the discrimination diagram of Mn-(Cu + Co + Ni) × 10-Fe (Figure 12).

(b) The siliceous rocks were deposited in a continental abyssal environment. The Sc/Th ratios of the siliceous rocks range from 0.10 to 13.85, which agree well with that of the siliceous rocks formed in the continental margin [61]. The U/Th ratios range from 0.07 to 4.91, which denote a depositional environment that was remote from the mainland [61]. The V/(V + Ni) ratios range from 0.09 to 0.72, which suggests that the deposition occurred in an oxygen-rich environment with V/(V + Ni) < 0.46 [64]. The Sr/Ba ratios range from 0.04 to 6.95 with an average of 0.95, which indicate an abyssal sea or stagnant shallow sea [65]. In the discrimination diagram of Ti-V, the siliceous rocks plot into the category of marginal sea (Figure 13).

(c) There was slight influence from the mafic magmatism. According to [64], the V/Cr > 2 and Ni/Co > 4 reflect an anoxic depositional environment, while the V/Cr < 2 and Ni/Co < 4 indicates an oxygen-rich depositional environment. The V/Cr ratios of the analysed siliceous rocks range from 0.25 to 1.11, which indicate that the depositional environment was oxygen-rich. The Ni/Co ratios range from 0.96 to 9.87, which indicate either an oxygen-rich depositional environment or a participation of mafic magmatism [64]. The presence of mafic magmatic activity could also contribute to the high Cr content in the siliceous rocks [66]. According to the Sc/Th, U/Th and Sr/Ba ratios, the V/Cr and Ni/Co ratios were probably influenced by the mafic magma rather than an aerobic environment. The associated magmatic activity should be related to the deep faults, such as Fengxian-Fengzhen-Shanyang and Jiudianliang-Zhenan-Banyan faults [1, 32, 34]. Although there was no volcanic activity during the deposition process (Figure 11), the deep faults in the Fengtai area could also have provided favorable conditions for the mafic magmatism to affect hydrothermal convection.

(3) The analytical results of the rare earth element are shown in Table 4, and they indicated the following.

(a) The siliceous rocks originated from hydrothermal precipitation with slight influences from terrigenous materials. The ΣREE values of the siliceous rocks range from 3.28 ppm to 19.75 ppm with an average of 9.83 ppm, which is consistent with that of siliceous rocks of hydrothermal sedimentary genesis [67]. Normalized by the PAAS [44] (Figures 14(a) and 14(b)), the siliceous rocks are divided into two groups. One group is tilted to the left with positive Eu anomalies and slightly weak Ce negative anomalies (Figure 14(a)), which is consistent with those of siliceous rocks with hydrothermal genesis. The other group is similar to that of the nonhydrothermal siliceous rock with negative Eu anomalies (Figure 14(b)) but does not support the nonhydrothermal genesis because of their inclination to the left. Because the ocean basin was insufficiently wide to prevent the terrestrial materials from influencing the original sedimentation process, the REE were only slightly affected by the terrestrial input with negative Eu anomalies (Figure 14(b)).

(b) The siliceous rocks were deposited in a marginal sea basin. The (La/Yb)_*N*_ ratios of the analysed siliceous rocks range from 0.10 to 1.52, similarly to that of siliceous rock deposited in the basin of the marginal sea [68]. The *δ*Ce values of siliceous rocks are typically 0.29 at the mid-ocean ridge, increasing gradually to 0.55 in the ocean basin, and range from 0.90 to 1.30 in the marginal sea next to the continent [68, 69]. In this study, the present *δ*Ce values (from 0.80 to 0.92) are consistent with those of siliceous rocks deposited in the basin of a marginal sea [69]. The (La/Ce)_*N*_ ratios of siliceous rocks are typically 1 when deposited in marginal seas [53], which reflect the influences of terrigenous input [70]. The (La/Ce)_*N*_ ratios of the analysed samples range from 0.85 to 1.46, which coincides with that of siliceous rock from marginal seas [53]. Also the (La/Lu)_*N*_ ratios (from 0.13 to 1.37) from the analysed siliceous rocks are approximately equal to that of siliceous rock deposited in marginal seas [67]. The hydrothermal diagenesis is represented by a Eu-positive anomaly [71], whose *δ*Eu value generally decreases from 1.35 at the mid-ocean ridge to 1.02 at 75 km from the mid-ocean ridge [53, 67]. The *δ*Eu values (from 0.28 to 1.84) of the analysed rocks did not match that of the siliceous rock formed at the mid-ocean ridge [69]. In the Al_2_O_3_/(Al_2_O_3_ + Fe_2_O_3_)-(La/Ce)_N_ discrimination diagram (Figure 15), the siliceous rocks plot in and next to the marginal sea field.

### 4.3. Raman Spectroscopy

**Figure 16:**
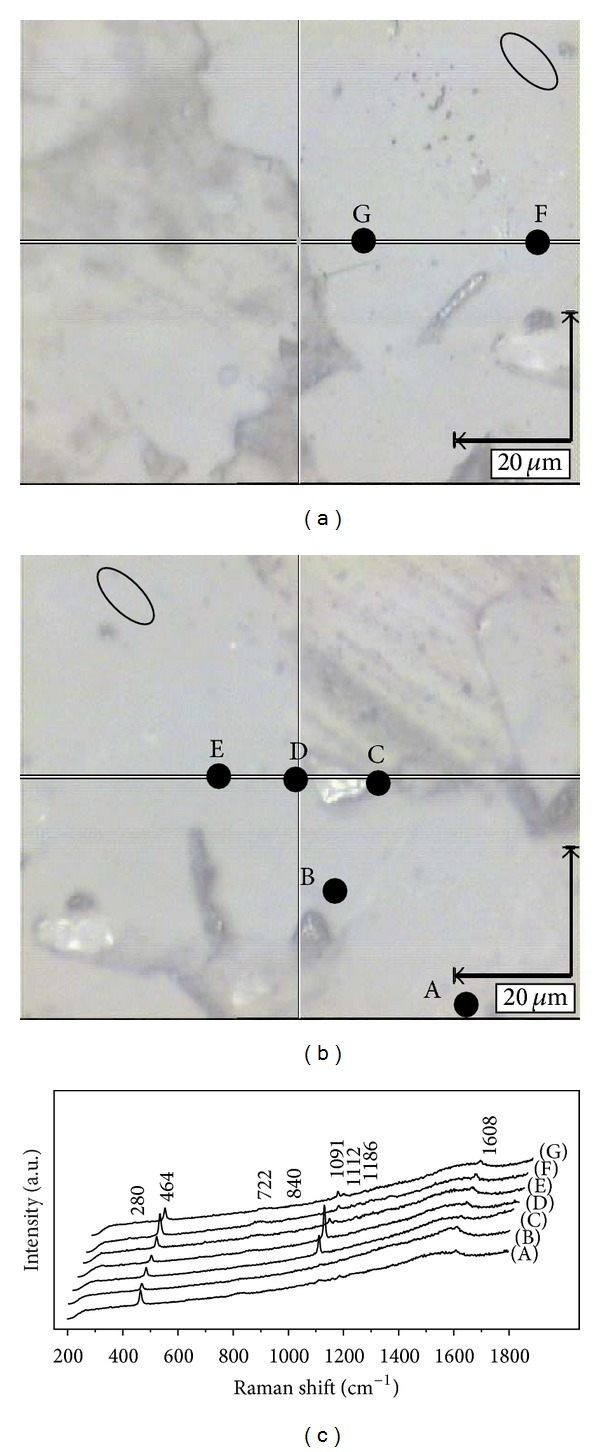
Points of Raman analysis for siliceous rocks of Bafangshan-Erlihe area. Microphotographs in Figures 16(a) and 16(b) under parallel nicols.

Raman analysis was performed on the points (A → G) as shown in the microphotographs of Figures 16(a) and 16(b). The micrographs illustrate deformed quartz grains and carbonate minerals. The ellipses in Figures 16(a) and 16(b) represent the straining ellipse for granular quartz formation, which was analysed in parallel (A, B and E) and oblique crossing (C to G) directions in relation to the macroaxis. In the Raman analysis, the points were analysed* in situ *in certain directions (the direction in parallel with points A, B, and E, while the oblique crossing direction including points C, D, E, F, and G). The spectral configurations for all the points are discussed elsewhere [15]. As shown in the spectrogram of the analysis points (A→G) (Figure 16(c)), the peaks are mainly caused by the Si–O bond of quartz and the CO_3_^2−^ ion of carbonate mineral. In Figure 16(c), the peaks at 464 cm^−1^ exhibit *α*-quartz according to previous work [72], and they were treated as a characteristic Raman shift. In addition, the peaks at 1112 cm^−1^ were also controlled by the Si–O bond according to previous studies [73–75]. There are remarkable scattering peaks at 1091 cm^−1^, and they fall into the overlapping range of the antisymmetric vibration peak (from 1010 cm^−1^ to 1125 cm^−1^) of Si–O and the R vibration peak of the V-band for CO_3_^2−^ (from 1050 cm^−1^ to 1090 cm^−1^). According to the Raman shift of calcite [76] and other carbonate minerals [77], the peaks at 1091 cm^−1^ should be attributed by the vibration of the V_1_-zone for the carbonate ions (CO_3_^2−^). Additionally, the peaks at 1091 cm^−1^ are in accordance with the weak peak of the V_4_-zone at 722 cm^−1^ for carbonate ions, which are similar to peaks of ankerite. In the spectrograms, two weak peaks at 840 cm^−1^ and 1186 cm^−1^ could have originated from the metamorphic reaction between silica and carbonate, which exhibit symmetric and antisymmetric stretching vibration peaks for Si–O–R, and they are too weak to accurately estimate. The peaks at 1608 cm^−1^, which are attributed to the C–C bond in benzene rings, came from the organic gum used in the experiment. The weak peaks at 280 cm^−1^ falling into the range of silicate mineral scattering peaks [78, 79] could have arisen from the metamorphic reaction between silica and carbonate. There are peaks on curves C to G for both quartz and carbonate minerals in the spectrogram (Figure 16(c)). This phenomenon demonstrates the carbonate composition infiltrating the quartz through the discontinuous portion caused by stress during orogeny. In addition, the degree of infiltration increases from the edge to the centre of the quartz grain [15].

**Figure 17:**
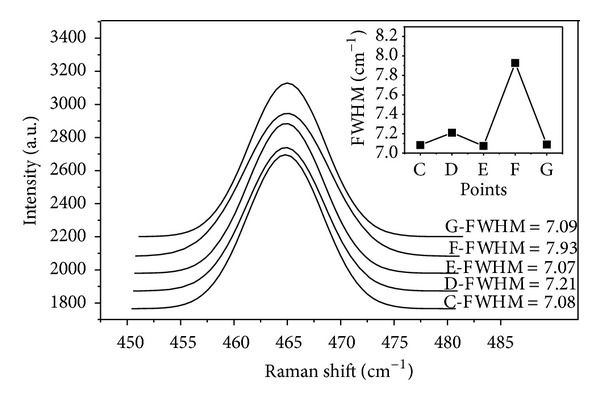
Gaussian fitting to characteristic Raman shift of quartz in siliceous rock from Bafangshan-Erlihe area.

The crystallinity and degree of order for the quartz grains increased during recrystallization [80, 81], which could be denoted by the Gaussian best-fit to the characteristic Raman peaks at 464 cm^−1^ for the quartz grains [82, 83].  Figure 17 suggests a Gaussian fitting for the characteristic Raman shifts at 464 cm^−1^ from points C to G. In the Gaussian fitting, the full width at half maximum (FWHM) values ascends from the centre outwards in concert with the crystallinity and degree of order but abruptly descends at the edge closest to the carbonate periphery. According to previous work [80], the crystallinity increases during the upper evolution of the quartz minerals from the centre to the quartz edge. Based on this finding, the FWHM values ascend from the centre outwards, meaning that the decline in crystallinity might be a result of the autorecrystallisation of quartz. The abrupt increase in crystallinity exists at the edge of quartz, and this should be contributed by the fluids during orogeny.

**Figure 18:**
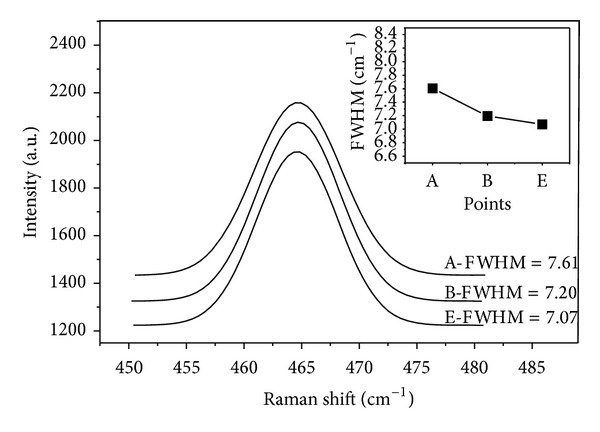
Gaussian fitting to a characteristic Raman shift for siliceous rock of the Bafangshan-Erlihe area.

According to Figure 18, the Gaussian fitting of characteristic Raman shifts at 464 cm^−1^ indicates discrepancies in a direction parallel to the macroaxis. In Figure 16(b), the points of A, B, and E lay parallel to the macroaxis of the quartz grains, and their FWHM values also showed some slight discrepancies. According to the discrepancy in the FWHM value, there are slight deviations of crystallinity parallel to the macroaxis of the straining ellipse. These deviations should relate to external factors during orogeny, such as the stress field and fluid effects. Therefore, there are overall geochemical stabilities for the siliceous rocks with slight changes in the crystallinity.

### 4.4. XRD

**Figure 19:**
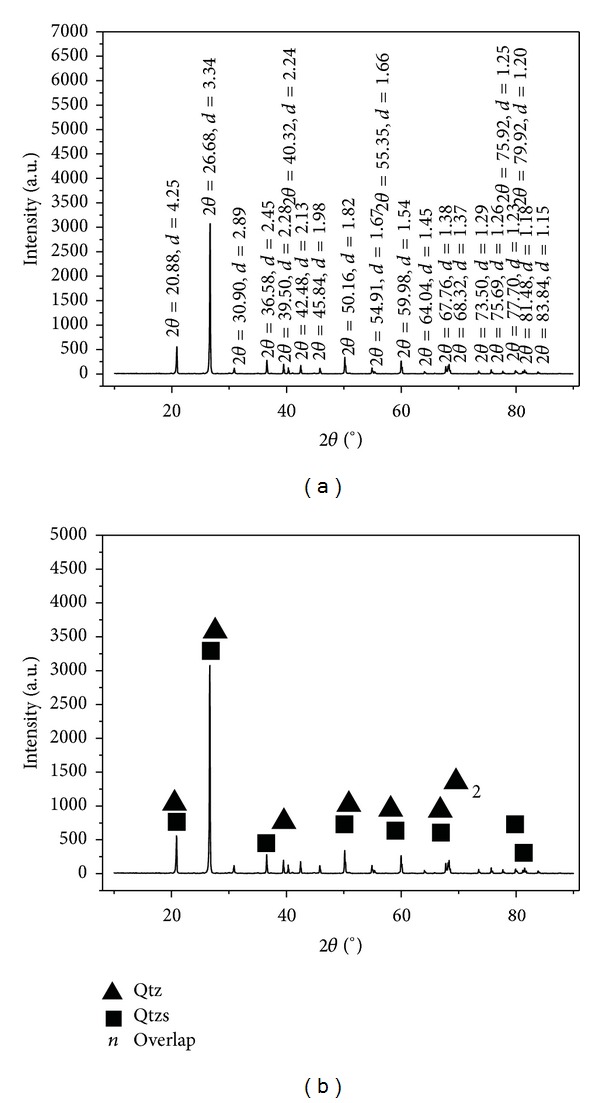
XRD diagram for siliceous rock of the Bafangshan-Erlihe area.

X-ray powder diffraction (Figures 19(a) and 19(b)) of the pure siliceous rock indicates quartz as the major mineral. Trace impurities, such as carbonate and clay minerals, are concealed in the analytical results. There are two types of quartz in the siliceous rocks (Figure 19(b)). One (Qtz) is hexagonal, with space group P3_1_21(152), the crystal cell parameters of which were *a* = *b* = 4.913 Å, *c* = 5.405 Å, and *Z* = 3, that is, identical to those of standard *α*-quartz. The other (Qtzs) is rhombohedral, having shorter crystal cell parameters and is similar to a quartz with space group P312(149), as noted elsewhere [15, 18]. The crystal cell parameters were *a* = *b* = 4.903 Å, *c* = 5.393 Å, and *Z* = 3.

The crystal cell parameters should be similar under uniform crystallizing environments and evolutionary processes. According to previous work, changes in crystal cell parameters can be effected by four factors as follows: temperature [84], stress [85], transformation into different crystal forms [86, 87], and isomorphous substitution [88]. In this study, crystal cell parameter shortening was possibly controlled by the primary factors of temperature and stress during the evolution of the COB, while the other factors could only have lengthened the cell parameters.

### 4.5. SEM

**Figure 20:**
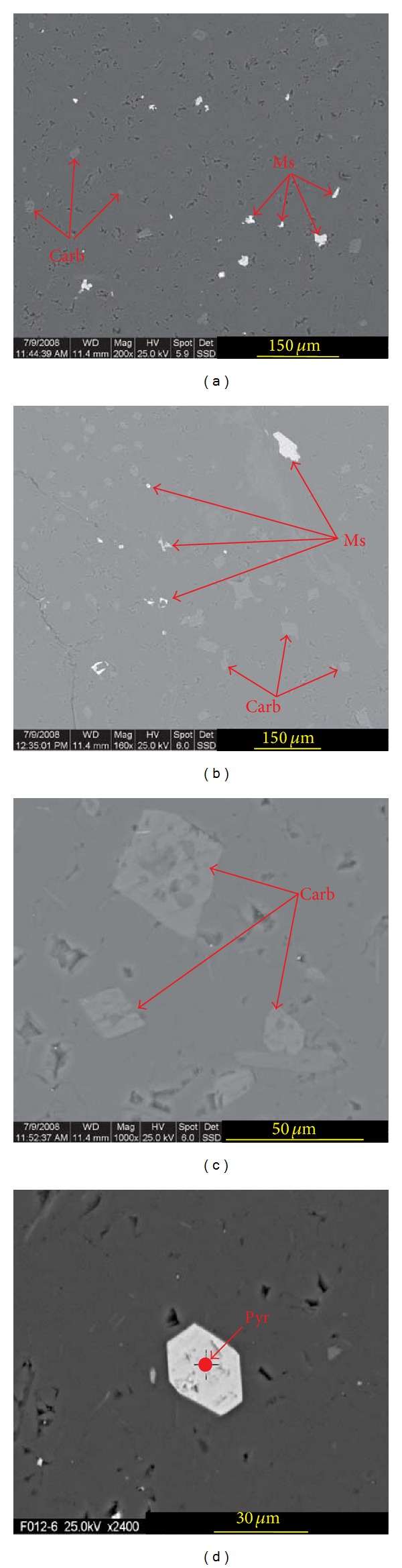
EBSD images for siliceous rock of the Bafangshan-Erlihe area (Carb: carbonate mineral, Ms: metal sulphide, Pyr: pyrite).

In the Electron Back Scatter Diffraction (EBSD) images of mineralized siliceous rock (Figure 20(a)), there are three principal phases, namely, quartz, carbonate mineral (calcite or dolomite), and metal sulphides (such as galena, sphalerite, or pyrite). The quartz particles of low crystallinity occupy the main body of the siliceous rocks, while the carbonates and metal sulphides are scattered with disseminated distribution (Figures 20(a) and 20(b)). The carbonate particles (Figure 20(c)) and pyrite (Figure 20(d)) sometimes show idiomorphic crystals, which indicates that they were originated from primary sedimentation. Metal sulphides were probably deposited at the end of high-temperature sedimentation. Although the siliceous rocks were involved in subsequent orogeny (Figure 20(b)), the metal sulphides are mainly disseminated without fracture-filling distribution (Figures 20(a) to 20(c)). Although the distribution of metal sulphides retained the primary characteristics of sedimentary genesis, a few metal sulphides, with fracture-filling distribution, might have been affected by the orogeny. These types of metal sulphides are distributed near the weaker part of the siliceous rocks, such as the fissures of the quartz and the carbonate mineral (Figure 20(b)). Therefore, it is suggested that the silica and metal sulphides were simultaneously deposited, and the metal sulphides are also precipitation products of hydrothermal sedimentation. The dominant minerals show low automorphism, which is attributed by their rapid deposition with insufficient time to crystallize and grow.

## 5. Discussion

### 5.1. Mineralogical and Geochemical Evolution

The studied siliceous rocks underwent mineralogical adjustments with maintenance of geochemical stability. In nature, elevated temperatures and pressures result in deformation of different rocks [89]. The quartz grains show plastic deformation under the strain rate of 0~10^−13^/s and temperature of 300°C [90]. Microscopically, we observed recrystallized quartz with larger grain size in the siliceous rocks without mineralization, which exhibits directional arrangement to some extent. In the Raman analysis, the FWHM values of characteristic peaks (next to 464 cm^−1^) showed inhomogeneity in the degree of crystallinity in diverse directions. This indicated that the crystallinity is different in the microareas of an individual quartz grain. As shown in the XRD analysis, the recrystallization of quartz was witnessed by the shortening cell parameter of quartz grains. Thus, the recrystallization of quartz is evidenced by both XRD analysis and Raman* in situ *analysis. Although the quartz underwent clear recrystallisation, under the influences of temperature and stress during the orogeny (according to [91]), these changes could only account for fabric changes. It is demonstrated that, the degrees of order in silica minerals may increase without exterior influence [76], and that geochemical stability of the total rocks can be still maintained with increasing crystallinity degree [80]. For the studied siliceous rocks, there is a high consistency to the hydrothermal genesis model, based on the geochemical and microfabric characteristics, as well as the geochemical discrimination diagrams of formation environment. Our study suggests that there is high stability for the original geochemical characteristics of the siliceous rocks and that these were maintained without any change in the total rocks.

### 5.2. Genesis of Siliceous Rocks

It is suggested here that the siliceous rocks in Central Orogenic Belt, China, and the Bafangshan-Erlihe ore districts are hydrothermal precipitates. The siliceous rocks, in addition to clay rock, clastic and carbonate rocks, are the most widely distributed sedimentary rock in this orogenic belt [3, 19, 92], and their formation is previously attributed to biogenesis [93], metasomatosis (or silicification) [94, 95] or chemical deposition [49, 96]. On a large scale, the sedimentary siliceous rocks could hardly be deposited in the marine environment with only terrigenous silica contribution. The high purity and large quantity of silica consumed for the deposition of the siliceous rocks, could not be completely caused by any terrigenous and nonhydrothermal deposition system [97–99]. This supports the idea that the massive sedimentary siliceous rocks were originated from hydrothermal precipitation according to [100]. The pure siliceous rocks could only have been deposited if the silica was present at a higher concentration in solution than other chemical components, resulting in high deposition rates of silica and favouring deposition of siliceous rocks instead of other sediments (carbonate, clay and metallic minerals) [16]. In this way, high rates of pure silica accumulation would develop without interference from other sediments. Terrigenous silica is difficult to precipitate during the migration process because of its very low solubility [101]. Even if there was rapid precipitation of silica at a low concentration, it was still difficult to deposit the siliceous sediments at a large scale with high purity after long-term and sustained transportation or other extreme conditions [99]. Furthermore, the SiO_2_ was extremely low in the normal seawater, and this could contribute to a low deposition rate [16, 97], while the quartz grains would grow better and bigger due to the adequate crystallizing time. The quartz grains in the siliceous rocks from the Bafangshan-Erlihe area, seem to have insufficient crystallization and growth, which is in contrast to quartz originated from the deposition of terrigenous silica with a low deposition rate. The microphotographs and EBSD indicate the silica minerals exhibited low-degree crystallinity, which was in accordance with the typical siliceous rocks of hydrothermal genesis [36]. During the hydrothermal precipitation, the quartz grains could not fully crystallise at high deposition rates of silica, and they therefore showed close-packed structures as a consequence of their rapid accumulation of the silica.

The ore-bearing siliceous rocks of Bafangshan-Erlihe area were considered to be of hydrothermal genesis also on the base of their geochemical characteristics. Some geochemical indices, such as the average Ba (196.64 ppm), ΣREE values (9.83 ppm) and ratios of Al/(Al + Fe + Mn) (0.36), Fe/Ti (335.44), (Fe + Mn)/Ti (347.80) and Ba/Sr (8.07), all suggest their genesis through hydrothermal deposition. In the geochemical discrimination diagrams, the siliceous rocks fall into the category of hydrothermal genesis and this is in agreement with their REE patterns. Therefore, it is considered that the siliceous rocks were deposited from a convective hydrothermal system within a crustal extensional setting. On the other hand, the MgO, ΣREE, Si/Al and U/Th of several samples disagree with the hydrothermal characteristics, and indicate that the sedimentary systems were affected by the input of nonhydrothermal materials (e.g., terrigenous and biological substances). The contribution of terrigenous materials is strongly supported by the presence of detrital zircons in the siliceous rocks [12]. Microorganisms, carrying terrigenous substances, were also involved in the precipitation process of the hydrothermal silica, and resulted in the observed fluctuations from normal hydrothermal characteristics. Therefore, the ore-bearing siliceous rocks in the Bafangshan-Erlihe area were originated from hydrothermal precipitation with some additional influence from terrigenous and biological sources.

### 5.3. Depositional Environment of Siliceous Rocks

The siliceous rocks of the Bafangshan-Erlihe area were deposited in a limited basin of a marginal sea. They were conformably deposited over the Middle Devonian marine limestone, which indicates their deposition in a marine environment with deep to shallower water. The geochemical indices, such as the Al/(Al + Fe + Mn), Al_2_O_3_/(Al_2_O_3_ + Fe_2_O_3_), Sc/Th, (La/Yb)_*N*_, (La/Ce)_*N*_, and (La/Lu)_*N*_, demonstrate that the siliceous rocks were deposited in a marginal sea basin, in coincidance with the geochemical discrimination diagrams. The K_2_O/Na_2_O, SiO_2_/(K_2_O + Na_2_O), and SiO_2_/Al_2_O_3_ ratios are significantly higher than those of volcanism-related siliceous rocks and indicate that there was no obvious volcanic activity during the formation process. However, magmatic bodies emplaced at depth and under extensional conditions, may have caused hydrothermal convection through deep faults, by providing heat in the system. The associated magma was mafic according to the V/Cr and Ni/Co ratios. The V/(V + Ni) ratios indicate that the sedimentation conditions were slighlty oxidative, which should reflect intermingling with terrigenous substances. In previous studies [102–104], it has been suggested that the ocean basin of the COB was width-limited and narrow, which strongly supports the input of terrigenous materials during the hydrothermal precipitation. In agreement with the previous studies [102, 104], a deposition of siliceous rocks in relatively shallow seawater, is also supported by the fact that these rocks are located on top of carbonate rocks of Gudaoling Formation. According to the geochemical characteristics and the presence of detrital zircons in the siliceous rocks [12], terrigenous materials participated in the sedimentation process of hydrothermal silica. The hydrothermal activity attracted many elements with an affinity to silicon [105], and this attraction might induce the biological production within a large population of organisms [106, 107]. These organisms can carry nonhydrothermal substances because of their long-distance activities and needs for special elements, such as phosphorus [108]. Under this situation, the organisms which were involved in the sedimentation process exhibit also terrigenous characteristics, and their dead bodies and catabolites have been deposited together with hydrothermal sediments according to [106]. Both terrigenous material and biological activities partly contributed to the formation of the siliceous rocks, as may be expected to be the case in a geological setting of a limited ocean basin [102–104]. By expanding previous studies that the COB was neritic facies during the Middle Permian [28], this work suggests that the whole COB was a limited ocean basin with deep to shallower seawater neritic facies since the Middle Paleozoic.

### 5.4. Hydrothermal Model

**Figure 21:**
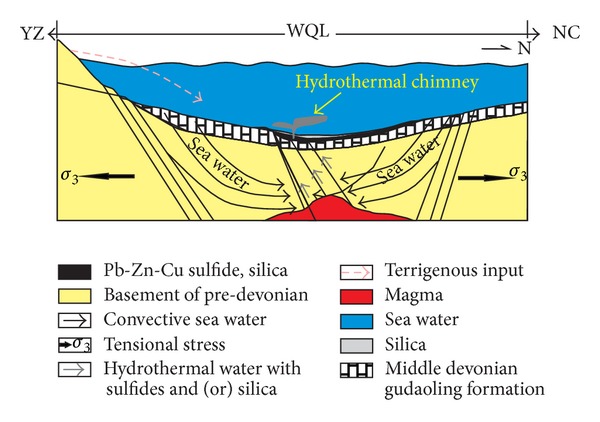
Hydrothermal precipitation model of the Bafangshan-Erlihe area (YZ: Yangtze block, WQL: western Qinling block, NC: North China block).

The hydrothermal sedimentation at the Bafangshan-Erlihe area was controlled by the extensional tectonic setting during Devonian times, and underwent different stages with diverse contribution of hydrothermal fluids (Figure 21). In general, the entire process of hydrothermal activity experienced an evolution from high-temperature precipitation of sulphide to low-temperature precipitation of silica (see below). Within the broad spectrum of exhalative-inhalative deposits, the two end-members, for example, sedimentary exhalative deposit (SEDEX) [109, 110] and volcanogenic massive sulphide deposit (VMS) [111, 112] are diverse in respect to their country rocks and ore-hosting rocks, as well as their fluid origin and characteristics. According to published literatures [113, 114], sulphide-bearing hydrothermal solution exhaled at the initial stages of hydrothermal activity under high temperatures, followed by exhalation of the silica-enriched hydrothermal waters, at a lower temperature with low dissolving capacity. Therefore, the silica rich hydrothermal water and sulphide-bearing hydrothermal solutions belonged to part of an evolving hydrothermal system. Previous studies delineate two models for the formation of SEDEX deposits: one considers tectonic activity and the geothermal gradient during sediment compaction (diagenesis) as the key factors for ore element migration in a highly saline formational brine, while the other considers hydrothermal fluids generated from seawater convection driven by a magma chamber intruding into sediments, and synsedimentary fault activity, as key factors in mineralization [115–118]. According to the V/Cr and Ni/Co ratios and discrimination diagrams, the siliceous rocks, as well as the metal sulphides, of the Bafangshan-Erlihe ore deposit were originated from the convection of hydrothermal water. Similarly, other trace elements could have been introduced from the hydrothermal water to form ore deposits (according to [8, 9]). In the Bafangshan-Erlihe region, the sea basin was formed as a result of the extensional tectonics (e.g. rifting). Normal basin-bounding faults related to the extensional tectonic setting near the suture zones could facilitate emplacement of magmas as proposed by [16]. The extensional tectonics could also provide a favorite environment to drive the hydrothermal convection [43]. The hydrothermal water moved upward along the syn-sedimentary fractures in the Bafangshan-Erlihe area, precipitating hydrothermal sediments on the seafloor within the basin after mixing with cold seawater.

During the final stages of magmatic activity, high temperature hydrothermal water with high potential solubility and dissolution of silica, were analogous to those precipitating modern metal sulphide chimneys (black smokers) [119]. In the ores from the Bafangshan-Erlihe Cu-Pb-Zn ore deposit [120], the isotopic ^206^Pb/^204^Pb (from 18.02 to 18.15), ^207^Pb/^204^Pb (from 15.56 to 15.81), and ^208^Pb/^204^Pb (from 37.99 to 38.66) are similar to those of modern oceanic sediments, which suggest their sources from both the upper crust and mantle. In addition, the *δ* ^34^S values of sulphides range from 6.03‰ to 11.86‰ and indicate a genesis of sulphides by sulphate reduction from sea water. These isotope geochemical characteristics strongly support the hypothesis that the ores were deposited in a marine context during hydrothermal convection, as also evidenced by the distribution of metal sulphides in the ore-bearing siliceous rocks. Furthermore, the orebodies were located at the bottom of the siliceous rocks, which suggests that their precipitation predates that of silica, and took place at the onset of the hydrothermal activity at higher temperatures.

A decrease in silica solubility at lower temperatures resulted in precipitation of pure silica. Since the solubility of silica is higher than that of metal sulphides at low temperatures [113], pure silica could only deposit at a relative low temperature. At this time, the hydrothermal precipitation process was akin to that of colder silica-enriched hydrothermal water (white chimney) [114]. Previous studies demonstrated that SiO_2_ solubility in the seawater at 150°C was 600 ppm [100], while it was ten times higher at 200°C than 50°C [121]. Therefore, the hydrothermal fluid was capable to dissolve silica and other trace elements from the sedimentary strata and became silica-enriched. By discharging on the seafloor, the silica-rich hydrothermal fluid mixed with the cold seawater, and the temperature dropped to cause silica precipitation as a consequence of SiO_2_ oversaturation [122].

The hydrothermal- and tectonic activities were contemporaneous at the Bafangshan-Erlihe area. Following the hydrothermal activity, deposition of the clastic rock formation (later metamorphosed to phyllites) and limestones took place. The hydrothermal system contributed to the precipitations of metal sulphide and siliceous rocks with geochemical characteristics of hydrothermal genesis. Terrigenous material, magmatism, and biological activity resulted in some geochemical deviation compared with classic hydrothermal sediments, but without changing the hydrothermal characteristics of the whole sedimentary formation.

## 6. Conclusions

(1) Siliceous rocks of the Bafangshan-Erlihe ore deposit were originated from hydrothermal precipitation. In microphotographs and EBSD images, the low degree of crystallinity and close-packed quartz grain texture indicates their hydrothermal genesis. The SiO_2_ content varies from 71.08% to 95.30% with an average of 84.10%. The hydrothermal genesis of the siliceous rocks is witnessed by the Al/(Al + Fe + Mn) ratio ranging from 0.03 to 0.58, (Fe + Mn)/Ti ranging from 15.59 to 1031.45, Ba ranging from 42.45 ppm to 503.00 ppm and ΣREE values ranging from 3.28 ppm to 19.75 ppm.

(2) Siliceous rocks of the Bafangshan-Erlihe region were deposited in marginal sea basin according to the geochemical discrimination diagrams. Additional geochemical data indicating that the deposition environment was a basin of a marginal sea, are Al/(Al + Fe + Mn) ratios ranging from 0.03 to 0.58, Sc/Th ratios ranging from 0.10 to 13.85, (La/Yb)_*N*_ ratios ranging from 0.10 to 1.52, (La/Ce)_*N*_ ratios ranging from 0.85 to 1.46 and (La/Lu)_*N*_ ratios ranging from 0.13 to 1.37.

(3) The siliceous rocks in the Central Orogenic Belt, China, had a periodic distribution in geological history and were mainly developed under periods of continental rifting. There were three depositing phases for the marine siliceous rocks with broader distribution from Mesoproterozoic to Jurassic. The periods for the positive peaks of distribution number were Mesoproterozoic, Cambrian ~ Ordovician, and Carboniferous ~ Permian. During the Caledonian (from Simian to Late Silurian) and Hercynian (from Devonian to Late Permian) periods, the siliceous rocks are widely distributed as a result of extentional tectonics, favoring their formation. The Jinning, Caledonian, Hercynian, Indosinian, and Yanshanian orogenies contributed to compressional setting and resulted in a sudden decrease in distribution numbers of siliceous rock.

(4) Hydrothermal fluids ascended along syn-sedimentary faults in the Bafangshan-Erlihe area, discharging hydrothermal sediments on the seafloor after mixing with cold seawater. The hydrothermal activity of the Bafangshan-Erlihe ore deposit evolved from initial high-temperature towards late low-temperature stages. High-temperatured hydrothermal sediments include metal sulphides and silica, while low-temperature sediments were mainly composed of silica. Apart from the hydrothermal sedimentation, terrigenous input, magmatism, and biological activity partly contributed to some geochemical indicators deviating from typical hydrothermal characteristics.

## Figures and Tables

**Table 1:** Major element analysis data (%) for siliceous rocks from Bafangshan-Erlihe area.

NO.	Description of samples	SiO_2_	TiO_2_	Al_2_O_3_	Fe_2_O_3_	FeO	MnO	MgO	CaO	Na_2_O	K_2_O	P_2_O_5_	LOI	Total
FE001	Siliceous rock without mineralization	71.08	0.00	0.54	0.19	1.08	0.08	0.88	13.84	0.01	0.07	0.06	12.13	99.96
FE002	Siliceous rock without mineralization	89.16	0.06	2.26	0.10	0.96	0.06	0.77	1.79	0.03	0.57	0.02	3.10	98.87
FE003	Siliceous rock without mineralization	81.13	0.01	0.57	0.23	2.38	0.10	1.47	6.25	0.01	0.11	0.03	7.49	99.78
FE004	Siliceous rock without mineralization	75.57	0.07	1.96	0.28	3.88	0.12	1.89	5.86	0.03	0.57	0.03	8.85	99.11
FE005	Siliceous rock without mineralization	75.64	0.00	0.19	0.49	4.07	0.13	2.25	6.86	0.01	0.05	0.00	9.75	99.43
FE006	Siliceous rock without mineralization	74.56	0.22	5.55	0.51	2.40	0.11	1.35	6.06	0.07	1.61	0.06	7.48	99.97
FE007	Siliceous rock without mineralization	83.08	0.06	1.73	0.58	2.64	0.08	1.17	4.09	0.02	0.45	0.02	5.76	99.68
FE008	Siliceous rock without mineralization	95.30	0.05	0.98	0.06	1.18	0.12	0.28	1.17	0.15	0.10	0.01	0.59	99.97
FB001	Siliceous rock without mineralization	82.40	0.07	2.88	0.18	2.18	0.10	2.88	2.56	0.04	0.85	0.05	4.64	98.83
FB002	Siliceous rock without mineralization	81.27	0.09	4.00	3.86	1.78	0.12	1.06	1.64	0.07	1.09	0.09	5.26	100.33
FB003	Siliceous rock without mineralization	86.88	0.03	1.23	0.17	2.33	0.12	1.18	3.28	0.02	0.32	0.06	4.96	100.58
FB004	Siliceous rock without mineralization	88.84	0.08	2.12	0.65	0.48	0.02	0.50	3.56	0.08	0.66	0.05	3.61	100.65
FB005	Siliceous rock without mineralization	81.92	0.13	3.87	2.46	1.85	0.05	1.33	3.46	0.07	1.08	0.04	4.54	100.80
FB006	Siliceous rock without mineralization	92.04	0.04	2.15	0.35	1.66	0.08	1.13	0.72	0.06	0.68	0.06	1.76	100.73

Aver.	—	84.10	0.07	2.18	0.69	1.83	0.09	1.14	4.01	0.05	0.59	0.04	5.15	99.94

**Samples of FB001 to FB006 are from literature [1].

**Table 2:** Major elements and related geochemical indices for siliceous rocks from the Bafangshan-Erlihe area.

NO.	Si/Al	MnO/TiO_2_	K_2_O/Na_2_O	SiO_2_/(K_2_O + N_2_O)	SiO_2_/Al_2_O_3_	Fe_2_O_3_/FeO	SiO_2_/MgO	Al/(Fe + Al + Mn)	Al/(Al + Fe)	Al_2_O_3_/(Al_2_O_3_ + Fe_2_O_3_)	Fe/Ti	(Fe + Mn)/Ti	Al_2_O_3_/TiO_2_
FE001	115.60	45.98	5.38	856.39	131.14	0.18	81.14	0.22	0.23	0.74	972.08	1031.45	324.94
FE002	34.85	0.96	19.62	149.10	39.54	0.11	115.79	0.58	0.59	0.96	22.09	23.32	36.54
FE003	125.68	7.17	8.62	649.03	142.58	0.09	55.23	0.13	0.13	0.72	250.88	260.14	42.64
FE004	34.02	1.72	19.62	126.38	38.60	0.07	39.94	0.24	0.24	0.88	78.29	80.51	28.63
FE005	354.65	78.52	8.77	1357.98	402.34	0.12	33.66	0.03	0.03	0.28	3503.66	3605.04	112.71
FE006	11.83	0.48	23.63	44.51	13.42	0.21	55.35	0.56	0.57	0.92	16.98	17.60	25.42
FE007	42.40	1.43	20.59	174.90	48.11	0.22	70.89	0.27	0.27	0.75	70.13	71.98	29.58
FE008	85.37	2.59	0.64	387.38	96.85	0.05	346.53	0.33	0.35	0.94	35.48	38.83	21.85
FB001	25.22	1.43	21.25	92.58	28.61	0.08	28.61	0.45	0.46	0.94	43.37	45.21	41.14
FB002	17.91	1.33	15.57	70.06	20.32	2.17	76.67	0.34	0.34	0.51	75.67	77.40	44.44
FB003	62.26	4.00	16.00	255.53	70.63	0.07	73.63	0.24	0.25	0.88	107.29	112.45	41.00
FB004	36.94	0.25	8.25	120.05	41.91	1.35	177.68	0.57	0.58	0.77	17.26	17.58	26.50
FB005	18.66	0.38	15.43	71.23	21.17	1.33	61.59	0.39	0.39	0.61	40.52	41.02	29.77
FB006	37.74	2.00	11.33	124.38	42.81	0.21	81.45	0.42	0.43	0.86	64.00	66.59	53.75

Aver.	54.27	5.28	13.81	269.67	61.56	0.44	136.70	0.36	0.37	0.82	132.05	138.87	56.20

**Samples FB001 to FB006 from literature [1].

**Table 3:** Trace element analysis result (×10^−6^) and related geochemical indices for siliceous rocks from the Bafangshan-Erlihe area.

NO.	Sc	Ti	V	Cr	Mn	Co	Ni	Cu	Zn	Ga	Ge	Rb	Sr	Zr	Nb	Cs	Ba	Hf	Ta	Pb	Th	U	Y	**Ti/V**	**V/Y**	**U/Th**	**Sc/Th**	**V/(V+Ni)**	**V/Cr**	**Ni/Co**	**Sr/Ba**
FE001	1.08	26.68	3.36	5.26	423.30	0.98	3.57	11.09	44.93	0.42	1.94	2.21	161.40	1.40	0.09	1.01	214.00	0.03	0.00	14.67	0.93	0.07	7.92	7.93	0.42	0.07	1.16	0.49	0.64	3.63	0.75
FE002	0.94	293.00	12.07	12.76	172.30	21.76	29.01	3.61	487.30	4.07	14.23	16.40	17.82	24.63	1.55	1.18	126.80	0.20	0.07	1635.20	0.99	0.21	0.82	24.28	14.79	0.21	0.94	0.29	0.95	1.33	0.14
FE003	0.08	93.10	3.81	15.46	748.10	1.96	8.48	7.84	66.58	0.45	6.07	4.50	61.67	26.25	1.09	0.71	214.20	0.11	0.03	25.45	0.30	0.11	1.86	24.42	2.05	0.36	0.25	0.31	0.25	4.32	0.29
FE004	1.60	431.20	15.38	17.03	398.50	17.53	24.35	111.40	7757.60	3.49	14.28	24.18	29.62	3.79	0.39	0.73	319.00	0.53	0.11	1035.20	1.43	1.53	2.20	28.04	6.99	1.07	1.12	0.39	0.90	1.39	0.09
FE005	1.66	101.40	12.16	10.95	1219.00	3.18	4.75	140.30	426.80	2.06	3.18	11.53	124.10	13.45	3.00	0.18	42.45	0.08	0.02	1953.40	0.12	0.17	10.09	8.34	1.21	1.43	13.85	0.72	1.11	1.50	2.92
FE006	0.05	59.72	8.90	8.96	203.40	1.29	12.70	4.76	12.43	0.30	0.04	2.09	356.00	10.58	0.80	0.64	51.23	0.06	0.01	48.70	0.24	1.20	3.40	6.71	2.62	4.91	0.20	0.41	0.99	9.87	6.95
FE007	0.91	260.20	10.71	11.36	496.00	17.29	16.68	157.60	3165.40	1.84	8.17	13.33	33.00	3.14	0.19	0.21	80.51	0.26	0.06	885.90	0.95	0.42	1.87	24.30	5.74	0.44	0.96	0.39	0.94	0.96	0.41
FE008	1.04	600.90	13.83	15.24	345.50	2.92	9.96	45.18	124.90	2.28	0.64	4.62	72.08	15.70	1.41	1.17	330.50	0.25	0.13	48.73	0.53	0.19	4.03	43.45	3.44	0.37	1.97	0.58	0.91	3.41	0.22
FE009	0.15	110.10	7.77	20.34	170.30	5.05	8.80	376.40	—	3.13	7.29	7.12	10.63	13.37	0.90	0.80	247.80	0.16	0.03	9865.80	0.82	0.96	0.49	14.17	15.76	1.17	0.18	0.47	0.38	1.74	0.04
FE010	1.47	312.70	13.05	23.67	489.40	43.48	48.74	7691.00	221.00	1.51	5.20	12.83	35.96	7.08	0.36	0.42	70.60	0.64	0.09	—	1.41	0.21	2.61	23.96	5.00	0.15	1.04	0.21	0.55	1.12	0.51
FE011	1.14	411.30	13.70	16.68	147.30	143.20	145.30	209.90	214.10	2.91	12.28	23.52	10.36	12.07	0.54	0.26	159.60	0.29	0.09	423.10	1.37	0.32	1.12	30.02	12.20	0.23	0.83	0.09	0.82	1.01	0.06
FE012	0.04	113.00	6.82	24.36	217.70	3.55	10.01	32.74	1134.10	1.25	8.17	6.61	19.37	10.12	1.00	2.39	503.00	0.21	0.03	235.50	0.42	0.39	0.81	16.58	8.37	0.93	0.10	0.41	0.28	2.82	0.04

Aver.	0.85	234.44	10.13	15.17	419.23	21.85	26.86	723.65	1241.38	1.97	6.79	10.75	77.67	11.80	0.94	0.81	196.64	0.23	0.06	1470.15	0.79	0.48	3.10	21.02	6.55	0.97	1.88	0.40	0.73	2.76	1.04

*—: outwith detection limits of the instrument.

**Table 4:** REE analysis results (×10^−6^) and related geochemical indices for siliceous rocks from the Bafangshan-Erlihe area.

No.	La	Ce	Pr	Nd	Sm	Eu	Gd	Tb	Dy	Y	Ho	Er	Tm	Yb	Lu	∑REE	*δ*Ce	*δ*Eu	(La/Ce)_*N*_	(La/Yb)_*N*_	(La/Lu)_*N*_
FE001	3.09	6.46	0.86	3.49	0.86	0.38	1.12	0.23	1.36	7.92	0.27	0.75	0.11	0.68	0.10	19.75	0.92	1.84	1.00	0.33	0.37
FE002	2.25	3.20	0.30	0.89	0.15	0.02	0.15	0.02	0.15	0.82	0.03	0.10	0.02	0.13	0.02	7.43	0.90	0.72	1.46	1.33	1.27
FE003	0.62	1.36	0.19	0.92	0.26	0.07	0.26	0.05	0.33	1.86	0.06	0.18	0.03	0.19	0.03	4.55	0.91	1.28	0.95	0.24	0.27
FE004	3.39	5.53	0.59	1.94	0.32	0.05	0.32	0.06	0.38	2.20	0.09	0.25	0.04	0.29	0.05	13.29	0.90	0.68	1.28	0.86	0.83
FE005	0.91	2.22	0.37	1.94	0.78	0.21	1.12	0.25	1.59	10.09	0.34	0.88	0.12	0.65	0.08	11.45	0.88	1.05	0.85	0.10	0.13
FE006	1.88	3.21	0.40	1.61	0.33	0.06	0.35	0.06	0.36	3.40	0.07	0.19	0.03	0.17	0.02	8.72	0.85	0.77	1.22	0.84	1.01
FE007	1.81	3.01	0.34	1.27	0.29	0.02	0.28	0.06	0.33	1.87	0.07	0.21	0.04	0.25	0.04	8.02	0.89	0.28	1.25	0.53	0.50
FE008	2.24	4.58	0.60	2.49	0.56	0.19	0.58	0.10	0.58	4.03	0.12	0.37	0.06	0.41	0.06	12.93	0.91	1.58	1.02	0.41	0.40
FE009	0.72	1.37	0.18	0.82	0.17	0.02	0.16	0.02	0.09	0.49	0.02	0.04	0.01	0.04	0.01	3.66	0.87	0.63	1.10	1.44	1.36
FE010	2.85	4.74	0.53	2.02	0.48	0.05	0.46	0.08	0.50	2.61	0.11	0.35	0.05	0.39	0.07	12.66	0.89	0.47	1.25	0.54	0.47
FE011	3.51	5.53	0.54	1.60	0.20	0.01	0.19	0.03	0.17	1.12	0.04	0.14	0.02	0.17	0.03	12.17	0.93	0.15	1.32	1.52	1.37
FE012	0.70	1.24	0.14	0.57	0.13	0.02	0.13	0.02	0.12	0.81	0.03	0.08	0.01	0.09	0.02	3.28	0.91	0.60	1.17	0.55	0.53

Aver.	2.00	3.54	0.42	1.63	0.38	0.09	0.43	0.08	0.50	3.10	0.10	0.29	0.04	0.29	0.04	9.83	0.90	0.84	1.16	0.72	0.71

–*N* represent normalized by PAAS [44], *δ*Ce, and *δ*Ce come from [45]: *δ*Ce = Ce/Ce∗ = Ce_*N*_/(La_*N*_ × Pr_*N*_)^1/2^ and *δ*Eu = Eu/Eu∗ = Eu_*N*_/(Sm_*N*_ × Gd_*N*_)^1/2^.
